# High-risk screening of late-onset Pompe disease: A different early portrait in China

**DOI:** 10.3389/fneur.2022.965207

**Published:** 2022-09-27

**Authors:** Kexin Jiao, Jihong Dong, Sushan Luo, Liqiang Yu, Qing Ke, Zhiqiang Wang, Xinghua Luan, Xiaojie Zhang, Junhong Guo, Yan Chen, Xihua Li, Song Tan, Fangyuan Qian, Jianming Jiang, Xuen Yu, Dongyue Yue, Changxia Liu, Lijun Luo, Jianping Li, Yanzhou Qu, Lan Chen, Jianglong Tu, Chong Sun, Chong Yan, Jie Song, Jianying Xi, Jie Lin, Jiahong Lu, Chongbo Zhao, Wenhua Zhu, Qi Fang

**Affiliations:** ^1^Department of Neurology, Huashan Hospital Fudan University, Shanghai, China; ^2^National Center for Neurological Disorders (NCND), Shanghai, China; ^3^Huashan Rare Disease Center, Shanghai Medical College, Huashan Hospital, Fudan University, Shanghai, China; ^4^Department of Neurology, Zhongshan Hospital, Fudan University, Shanghai, China; ^5^Department of Neurology, The First Affiliated Hospital of Soochow University, Shanghai, China; ^6^Department of Neurology, The First Affiliated Hospital, School of Medicine, Zhejiang University Hangzhou, Zhejiang, China; ^7^Department of Neurology, Institute of Neurology, First Affiliated Hospital, Fujian Medical University, Fuzhou, China; ^8^Department of Neurology, Shanghai Jiao Tong University Affiliated Sixth People's Hospital, Shanghai, China; ^9^Department of Neurology, First Hospital, Shanxi Medical University, Taiyuan, China; ^10^Department of Neurology, Tongji Hospital, Tongji University, Shanghai, China; ^11^Department of Neurology, Children's Hospital of Fudan University, Shanghai, China; ^12^Department of Neurology, School of Medicine, Sichuan Provincial People's Hospital, University of Electronic Science and Technology of China, Chengdu, China; ^13^Department of Neurology, School of Medicine, Affiliated ZhongDa Hospital, Research Institution of Neuropsychiatry, Southeast University, Nanjing, China; ^14^Department of Neurology, First Affiliated Hospital to Naval Medical University, Shanghai, China; ^15^Affiliated Hospital of the Institute of Neurology, Anhui University of Chinese Medicine, Hefei, China; ^16^Department of Neurology, Jing'an District Center Hospital of Shanghai, Fudan University, Shanghai, China; ^17^Department of Neurology, The Fourth Affiliated Hospital of Nantong University, Yancheng, China; ^18^Department of Neurology, Wuhan No.1 Hospital, Wuhan, China; ^19^Department of Geriatrics, School of Medicine, Renji Hospital, Shanghai Jiao Tong University, Shanghai, China; ^20^Department of Neurology, The Sixth Affiliated Hospital of Wenzhou Medical University, Lishui, China; ^21^Department of Neurology, Nantong first people's Hospital, Nantong, Jiangsu, China; ^22^Department of Neurology, The Second Affiliated Hospital of Nanchang University, Nanchang, China

**Keywords:** late-onset Pompe disease, respiratory failure, high-risk screening, tandem mass spectrometry, dried blood spot

## Abstract

**Introduction:**

The lack of knowledge regarding the differences between Chinese and other ethnicities in the early manifestation of late-onset Pompe disease (LOPD) prohibits the development of an effective screening strategy. We conducted a multicenter screening study to determine LOPD prevalence in high-risk populations and define the early manifestation of LOPD in China.

**Methods:**

Between August 2020 and April 2021, the participants were prospectively identified through medical examination at 20 centers from inpatient departments and outpatient neuromuscular clinics in China. The inclusion criteria were as follows: (1) age ≥ 1 year and (2) either one of the following conditions: (a) persistent hyperCKemia, (b) muscle weakness of the axial and/or limb-girdle muscles, or (c) unexplained restrictive respiratory insufficiency (RI). Enzymatic activity of acid α-glucosidase (GAA) was measured in a dried blood spot (DBS) using a tandem mass spectrometry (MS/MS) assay. Next-generation sequencing (NGS) was used to evaluate all samples with decreased GAA activity, searching for *GAA* mutations and pseudodeficiency alleles.

**Results:**

Among the 492 cases, 26 positive samples (5.3%) were detected in the DBS test. Molecular studies confirmed a diagnosis of LOPD in eight cases (1.6%). Using MS/MS assay, GAA activities in individuals with pseudodeficiency could be distinguished from those in patients with LOPD. The median interval from the onset of symptoms to diagnosis was 5 years. All patients also showed RI, with a mean forced vital capacity (FVC) of 48%, in addition to axial/proximal muscle weakness. The creatine kinase (CK) level ranged from normal to no more than 5-fold the upper normal limit (UNL). LOPD with isolated hyperCKemia was not identified.

**Conclusion:**

Less frequent hyperCKemia and predominant RI depict a different early portrait of adult Chinese patients with LOPD. A modified high-risk screening strategy should be proposed for the early diagnosis of Chinese patients with LOPD.

## Introduction

Pompe disease, also known as glycogen storage disease type 2 or acid maltase deficiency, is a multisystemic metabolic disease caused by lysosomal acid α-1,4-glucosidase (GAA) deficiency (1). The age at onset and phenotype are variables in late-onset Pompe disease (LOPD). Most patients with LOPD exhibit axial and proximal skeletal muscle weakness, and morbidity and mortality are associated with respiratory insufficiency (RI) (2). Sufficient evidence suggests that enzyme replacement therapy (ERT) is more effective when administered early, before extensive and possible irreversible muscle damage occurs. This emphasizes the importance of early diagnosis (3). However, patients with LOPD exhibit various symptoms, such as pauci-neuromuscular symptoms at onset; hence, the diagnosis can easily be overlooked (4, 5). Patients with LOPD are typically diagnosed 144 (12–480) months after the onset of the first symptom (6). The mean delay in LOPD diagnosis in mainland China is 7.2 years (7).

Rapid diagnostic techniques, such as the dried blood spot (DBS) test, have been widely used for detecting GAA activity in large-scale screening studies to enable early LOPD diagnosis. The importance of DBS for Pompe disease screening in patients with unclassified limb-girdle weakness or asymptomatic hyperCKemia, with a prevalence of 1.1–4.6%, has been demonstrated in several studies (8–15). GAA mutational hotspots between Chinese and European patients with LOPD show variations (16). Chinese patients with LOPD appear to have a more severe phenotype than patients globally, particularly regarding respiratory involvement and the need for mechanical ventilation (7, 17). Unfortunately, no study has investigated high-risk LOPD screening in the Chinese population.

Herein, we conducted a prospective, multicenter observational study to identify undiagnosed LOPD in a large, high-risk population to determine the appropriate screening criteria for Chinese patients with LOPD.

## Methods

### Study design and patient recruitment

Between August 2020 and April 2021, the participants were prospectively identified through medical examinations at 20 medical centers from inpatient departments and outpatient neuromuscular clinics in China. The inclusion criteria were as follows: (1) patients aged ≥1 year; and (2) patients having at least one of the following conditions: (a) unexplained persistent hyperCKemia, (b) axial/limb-girdle muscular weakness, or (c) unexplained RI. Serum creatine kinase (CK) levels >1.5-fold the upper normal limit (UNL) that manifested at least two times within a month were considered as a sign of persistent hyperCKemia. Axial/limb-girdle muscular weakness was defined as trunk and proximal arm and leg muscle weakness, including the paravertebral muscles, especially the neck flexors and shoulder, upper arm, pelvic, and thigh muscles. The development of respiratory symptoms (dyspnea at rest, exertional dyspnea, orthopnea, hypersomnolence, or headache on awakening), hypercapnia, or abnormal pulmonary test results suggesting neuromuscular weakness were all considered signs of RI. Patients with spastic paralysis or those with family members diagnosed as having LOPD were excluded from the study.

### Sampling and tandem mass spectrometry (MS/MS) assay

Blood samples were collected from peripheral veins and immediately spotted onto filter papers. All samples were anonymized and analyzed at Suzhou PerkinElmer Medical Laboratory, China. GAA activity was analyzed using MS/MS assay as previously reported (18, 19).

### Gene sequencing

If the GAA activity of the DBS tests was below 1.46 μmol/L/h, next-generation sequencing (NGS) was performed. Using a genomic DNA extraction kit (Tiangen, China), genomic deoxyribonucleic acid (DNA) was extracted from the peripheral blood leukocytes. Targeted NGS covering 603 genes related to inherited muscular disorders and subsequent Sanger confirmation was performed for 26 patients. The Human Gene Mutation Database was used to address the novel variants. The predicted severity for each known mutation was based on the information obtained from the Pompe mutation database maintained by Erasmus Medical Center (www.pompecenter.nl). The American College of Medical Genetics and Genomics (ACMG) guidelines were used to interpret and categorize the novel variants (20).

### Statistical analysis

The differences in patient characteristics, along different levels, were tested for statistical significance using the chi-square or Fisher's exact tests for compiling categorical data; continuous variables were determined using student's *t*-test or the Mann-Whitney test. Differences in frequency were compared using the chi-square test or Fisher's exact test as appropriate. SPSS statistical software (version 27.0; SPSS; Chicago, IL, USA) and GraphPad Prism 9 were used to analyze the test data. All *p*-values were two-tailed, and statistical significance was set at *p* < 0.05.

### Standard protocol approvals, registrations, and patient consents

All patients provided verbal and written consent before blood sampling. This study was conducted in accordance with the ethical principles of the Declaration of Helsinki. The study was approved by the Ethics Committee of Huashan Hospital and the participating centers.

## Results

### Demographic and clinical findings

The study included 492 patients aged 1–86 years with a median of 42 years (male:female, 1.2:1). The demographic and clinical characteristics of the cohort are summarized in Table 1.

**Table 1:** Summary of the demographic and clinical characteristics of the participants.

	**Patients (*n* = 492)**	
Age (years, mean ± SD, range)	42 ± 19.1, 1-86	≤ 14 years, 39 (7.9%) >14 years, 453 (92.1%)
Sex (Male:Female)	264:228	
Inclusion criteria		
HyperCKemia	251 (51.0%)	Isolated, 66 (13.4%) With LGMW/AW, 164 (33.3%) With RI, 3 (0.6%) With LGMW/AW and RI, 18 (3.7%)
LGMW/AW	413 (83.9%)	Isolated, 214 (43.5%) With hyperCKemia, 167 (33.9%) With RI, 13 (2.6%) With LGMW/AW and RI, 19 (3.9%)
RI	40 (8.1%)	Isolated, 5 (1.0%) With LGMW/AW, 13 (2.6%) With hyperCKemia, 3 (0.6%) With LGMW/AW and hyperCKemia, 19 (3.9%)

AW, axial muscle weakness; LGMW, limb-girdle muscle weakness; RI, respiratory insufficiency; SD, standard deviation.

**Figure 1:**
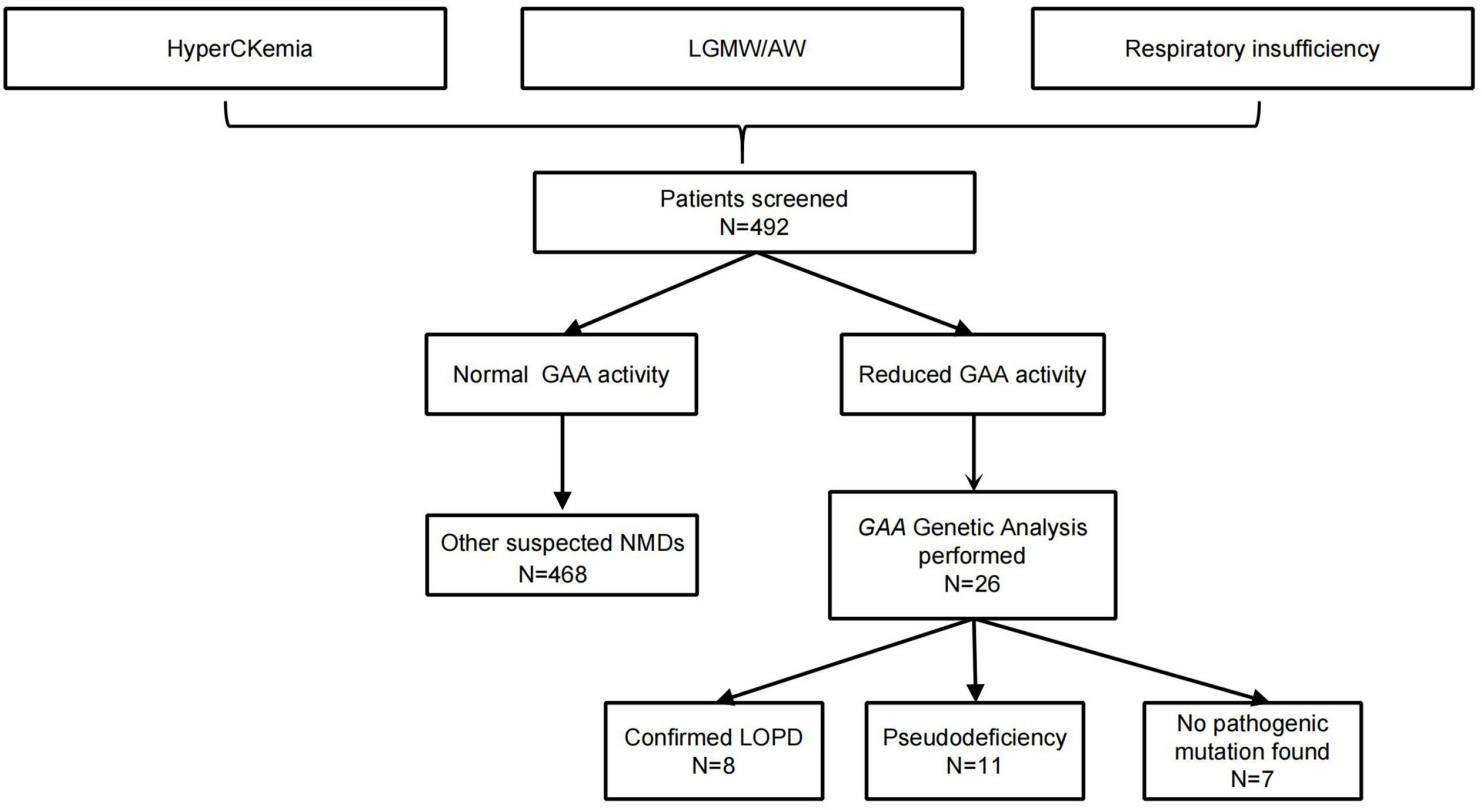
A flow diagram of the study design and analysis. AW, axial muscle weakness; GAA, acid α-1,4-glucosidase; LGMW, limb-girdle muscle weakness; LOPD, late-onset Pompe disease; NMDs, neuromuscular disorders.

Lysosomal acid α-1,4-glucosidase activity results were within the normal range in 466 patients (94.7%). Twenty-six patients with a median age of 38.5 years had decreased GAA activity below the cutoff (1.46 μmol/L/h). Twenty-two (4.5%) patients had axial/limb-girdle muscle weakness, 12 (2.4%) patients had hyperCKemia, and five (1.0%) patients had RI. Eight patients (1.6%) were diagnosed with LOPD using mutational analysis (Figure 1). The c.2238G>C (p.W746C) mutation was found in 62.5% of patients (5/8) at an allele frequency of 43.8% (7/16), making it the most frequent mutation. A novel variant, c.568C>G (p.R190G), was detected *in trans* with a known pathogenic variant in one patient (patient 1), which was classified as “pathogenic” according to the ACMG guidelines (PS3, PM1, PM2, PM3, PM5, PP1, and PP3).

The 18 other patients underwent comprehensive clinical, electrophysiology, and genetic studies. An alternative diagnosis, including facioscapulohumeral dystrophy (*n* = 2), statin-induced myopathy (*n* = 1), myofibrillar myopathy (*n* = 1), spinal muscular atrophy type 3 (*n* = 1), amyotrophic lateral sclerosis (*n* = 2), paraneoplastic syndrome (*n* = 1), and peripheral neuropathy (*n* = 1), was established in nine patients (Supplementary Table S1).

### Clinical and laboratory features of the eight patients newly diagnosed with LOPD

**Figure 2:**
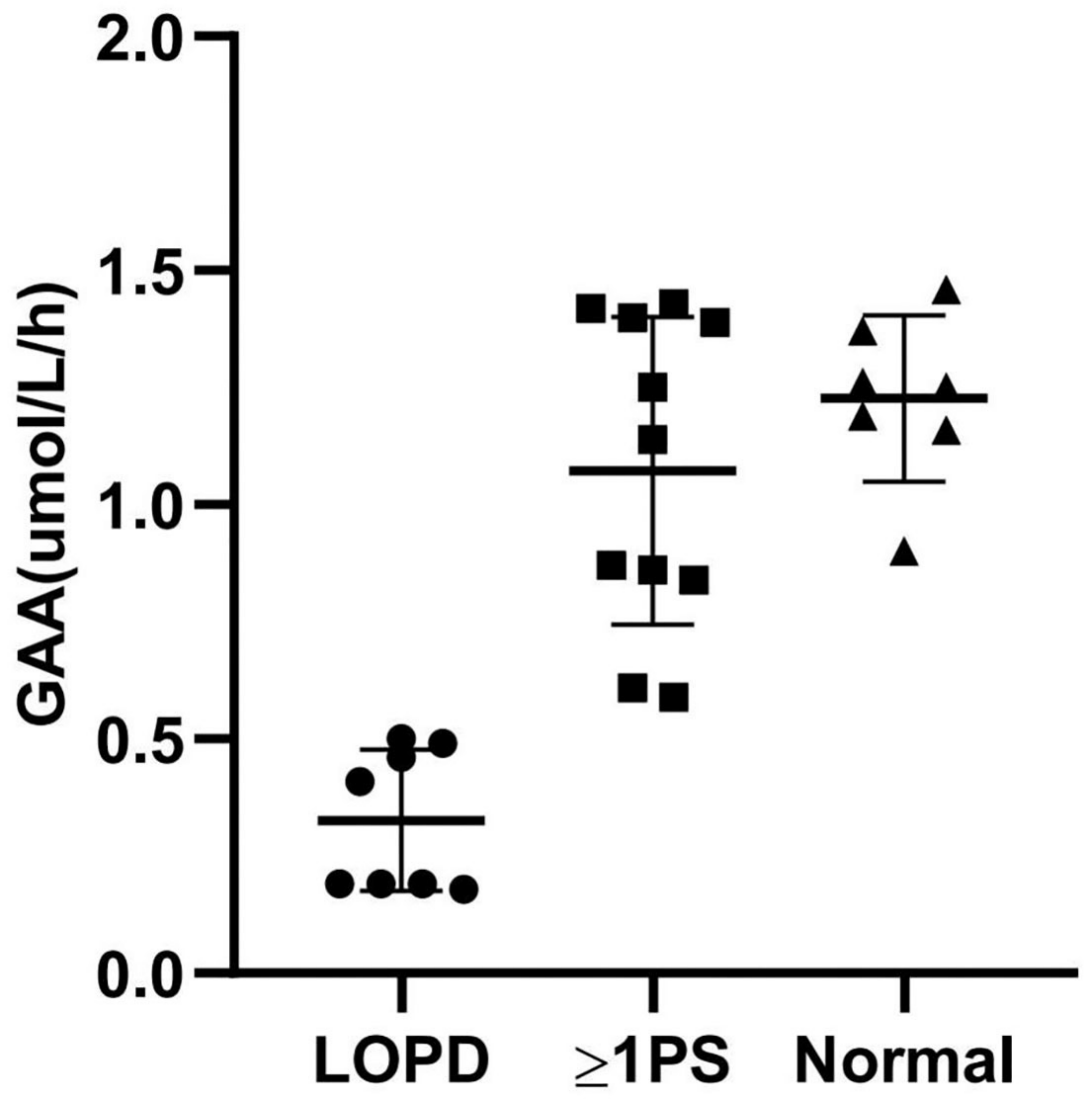
Correlation between GAA activity and genotype. GAA, acid α-1,4-glucosidase; LOPD, late-onset Pompe Disease; PS, pseudodeficiency with or without GAA mutations; Normal, normal controls.

An average GAA activity of 0.33 μmol/L/h was observed in the eight patients. Among the rest with decreased GAA activity in the DBS test, 11 patients (42.3%) carried at least one pseudodeficiency allele (c.[1726A; 2065A]). The individual GAA activities, genotypes, and CK levels are presented in Supplementary Table S1. When measured with MS/MS, the pseudodeficiency DBS results were distinguished from the LOPD samples (Figure 2). Mildly decreased GAA activity (1.23 μmol/L/h on average) was observed in seven patients without GAA mutations or pseudodeficiency alleles. We assumed that poor sampling or shipment may have contributed to the false positive result because other enzymes, such as acid β-glucocerebrosidase for Gaucher, acid α-galactosidase A for Fabry, and acid α-L-iduronidase for mucopolysaccharidosis type I (MPS-I), tested in the same multiplex assay were also typically mildly decreased in these patients. A second DBS test was not performed.

In total, 5 of the eight newly diagnosed patients with LOPD were women (Table 2). The median age at onset was 33.5 ± 10.8 years, while the median age at diagnosis was 41.0 ± 7.4 years. The median time from disease onset to the final diagnosis was 5 years. Five patients were presented with hyperCKemia, while two patients (25%) had a CK level of >2-fold the UNL. None of the patients had isolated hyperCKemia. Axial/proximal muscle weakness was the characteristic feature of LOPD, which was observed in all eight patients. All except one patient had respiratory symptoms, such as exertional dyspnea, dyspnea at rest, or hypercapnia. One patient (patient 3) was presented with exertional dyspnea, while limb-girdle muscle strength was well preserved, and biceps biopsy revealed no vacuolar pathology (not shown). According to the predicted forced vital capacity (FVC) percentage in the upright position (FVC-U) and blood gas test, respiratory involvement was demonstrated in all with a mean of 48 ± 17% of the predicted FVC (range 21–70%). At the time of diagnosis or shortly thereafter, six of the eight patients required nocturnal non-invasive ventilation. Five patients experienced acute physiological decompensation, resulting in at least one admission to the intensive care unit before the final diagnosis.

**Table 2:** The clinical characteristics and molecular analysis of eight newly diagnosed patients with LOPD.

**Pt**	**Sex**	**BMI**	**Age at onset/ Dx (y)**	**Dx Delay (y)**	**Family history**	**Initial symptoms**	**CK/ UNL**	**Muscle strength upon examination (MRC)**							**Respiratory** ** insufficiency (FVC-U, %Pred)**	**NNV start (y)**	**ICU**	**ERT start (y)**	**GAA activity (μmol/** **L/h)**	***GAA* Muta** **tions** ** (NM_** **000** **152.4)**	**Pseudo-deficiency allele**
								**NF**	**SA**	**EF**	**HE**	**HF**	**KE**	**KF**							
1	M	22	31/51	20	+	Waddling gait	<1.5	2	5	4+	5-	5	5	5	No complaint (70)	NA	N	N	0.18	c.568C>G (R190G) ^*^/ c.1082C>T (P361L)	c.1726G/G, c.2065G/G
2	F	17	36/43	7	+	LL	<1.5	2+	5-	5	3-	2	5-	5-	Y (59)	42.5	Y	43.5	0.19	c.2238G>C (W746C)/ c.2238G>C (W746C)	c.1726A/A, c.2065A/A
3	F	18	43/44	1	?	Exertional dyspnea	<1.5	2	5	5	5	5	5	5	Y (39)	43.5	Y	44	0.19	c.2238G>C (W746C)/ c.2238G>C (W746C)	c.1726A/A, c.2065A/A
4	M	17	14/39	25	+	LL	1.6	3	4	4	2	2	4	4	Y (44)	39	N	N	0.5	c.953T>A (M318K)/ c.953T>A (M318K)	c.1726G/G, c.2065G/G
5	F	16	20/33	13	?	LL	3.9	2	4+	4	4+	2	5	5	Y (21)	28	Y	N	0.19	c.2238G>C (W746C)/ c.2024_ 2026del (N675del)	c.1726G/A, c.2065G/A
6	F	16	37/37.5	0.5	+	LL	2.7	2	5	5	3+	4-	5	5	Y (56)	37.5	Y	38	0.49	c.2238G>C (W746C)/ c.1799G>A (R600H)	c.1726G/A, c.2065G/A
7	M	24	45/48	3	?	Dyspnea	1.5	5-	4-	5	4-	5-	5-	5-	Y (ND)	NA	N	N	0.41	c.953T>A (M318K)/ c.953T>A (M318K)	c.1726G/G, c.2065G/G
8	F	14	29/29.5	0.5	?	LL	1.7	2	4	5	4-	3+	5	5	Y (NA)^?^	29	Y	N	0.46	c.2238G>C (W746C)/ c.2662G>T (E888^*^)	c.1726G/A, c.2065G/A

BMI, body mass index; CK, creatine kinase; Dx, diagnosis; EF: elbow flexors; ERT: enzyme replacement treatment; F, female; FVC-U, forced vital capacity percentage in the upright position; GAA, acid α-1,4-glucosidase; HE: hip extensors; HF: hip flexors; ICU: had experienced acute physiological decompensation and intensive care; KE: knee extensors; KF: knee flexors; LL, lower limb muscle weakness; M, male; MRC: Medical Research Council; N: no; NA, not applicable; ND, not done; NF: Neck flexors; NNV, nocturnal non-invasive ventilation; Pred, prediction; Pt, patient; SA: shoulder abductors; UNL, upper normal limit; Y, yes.

* A novel GAA variant c.568C>G (R190G) is classified as a pathogenic variant based on the ACMG standard (PS3, PM1, PM2, PM3, PM5, PP1, and PP3).

?Not applicable due to tracheotomy.

### Treatment

Shortly after the diagnosis was established, three patients received ERT. The median age at the start of ERT was 43.5 (38–44) years. After receiving ERT for a year, all three patients showed improvements on the 6-min walking test and the manual muscle test. After a year, patient 2's FVC-U was marginally improved. Patient 6 reported less dyspnea in the supine position, although the FVC-U remained unchanged. Despite the lack of FVC data, patient 3 reported a reduced time on the ventilator. The quality of life was improved in three patients.

## Discussion

This is the first prospective cohort for screening LOPD in a high-risk Chinese population. Herein, only 7.9% of the patients screened were aged <14 years since most centers involved were adult neurology departments. The prevalence of Pompe disease was 1.6% in adult patients with unclassified axial or limb-girdle muscle weakness, hyperCKemia, or RI. This finding is similar to those of previous studies, with a combined prevalence of 2.4% (Table 3) (8–15). The age at symptom onset and diagnosis was later than previous reports of Chinese LOPD cohorts (16, 17, 21, 22). Thus, this study expanded our understanding of the wide variation in the clinical spectrum of LOPD in China. The limitation of this study is that due to insufficient screening in a high-risk juvenile population, the prevalence might be underestimated and the early features of Chinese LOPD identified in this study could only be applied to adults.

**Table 3:** High-risk screening studies in late-onset Pompe disease.

**Year of publication**	**Country**	**Inclusion criteria**	**No. of investigated patients**	**Patients DBS positive**	**Patients with confirmed Pompe**	**LOPD prevalence (%)**	**HyperCKemia (%)**	**LGMW/AW (%)**	**Respiratory insufficiency (%)**
Present study	China	LGMW/AW, hyperCKemia, RI	492	26	8	1.6	5 (62.5)	8 (100)	8 (100)
2019 (8)	Poland	LGMW, hyperCKemia, RSS, dyspnea, myalgia	337	18	10	3.0	10 (100)	7 (70)	1 (10) ^***^
2018 (9)	Turkey	>18 years, undiagnosed myopathic syndrome	350	21	4	1.1	0	4 (100)	0 (0) ^**^
2017 (10)	Korea	Proximal muscle weakness, axial muscle weakness, lingual weakness, RI, hyperCKemia	90	16	2	2.2	2 (100)	2 (100)	1 (50)
2017 (11)	Portugal	LGMW, hyperCKemia±hypotonia	99	4	4	4.0	4 (100)	4 (100)	2 (50)
2016 (12)	Germany and UK	LGMW, unexplained hyperCKemia	3076	232	74	2.4	74 (100) ^**^	63 (85.1)	47 (63.7) ^*^
2015 (13)	Spain	>18 years, LGMW, asymptomatic or pauci-symptomatic hyperCKemia	348	20	16	4.6^*^	12 (75)	14 (87.5)	10 (62.5)
2015 (14)	Italy	>5 years, hyperCKemia, LGMW	1051	30	17	1.6	15 (94)	11 (65.05)	14 (82)
2017 (15)	Denmark	Unclassified LGMW, hyperCKemia, unexplained myopathy on muscle biopsy, unexplained RI, unspecified myopathy	103	3	3	2.9	3 (100)	3 (100)	0 (0) ^**^

DBS, dried blood spot; LOPD, late-onset Pompe disease; AW, axial muscle weakness; LGMW, limb-girdle muscle weakness; RI, respiratory insufficiency; RSS, rigid spine syndrome; UNL, upper normal limit;

* p < 0.05;

** p < 0.01;

*** p < 0.001.

The efficacy of DBS has been described in studies based on newborn screening and other studies on Pompe disease (8–15). In the Asian population, the pseudodeficiency variant c.[1726G>A; 2065G>A] (p.[G576S; E689K]), which lowers GAA activity to the range observed in Pompe disease, has been frequently identified (23). Pompe disease screening was complicated due to the high prevalence of the pseudodeficiency allele (10, 24–26). We opted for direct NGS over a repeat DBS test as the second test to confirm the diagnosis because a newborn screening study in Taiwan revealed that MS/MS could distinguish between affected and pseudodeficiency patients (26). In the study, 11 of the 18 false positive samples carried at least one pseudodeficiency allele. MS/MS again helped to distinguish between patients with LOPD and carriers of pseudodeficiency alleles. Incorrect DBS spotting and sampling and environmental circumstances, such as a high temperature during transport, can result in deficient GAA activity. Careful clinical re-evaluation of these patients is warranted to establish an alternative diagnosis.

HyperCKemia is common in patients with inherited myopathy. CK level usually decreases with disease progression and the loss of muscle mass. In our cohort, the serum CK levels in the patients newly diagnosed with LOPD were generally low regardless of the disease course. Five out of eight patients were presented with a CK level of >1.5-fold the UNL, with only two patients having a CK level of >2-fold the UNL. None of the patients with “asymptomatic hyperCKemia” had LOPD. In our experience, a subset of adult Chinese patients with LOPD had consistently low serum CK levels throughout the disease course. In contrast, in a Spanish cohort, CK was increased in 13 of the 16 patients, with values ranging between two and eight times the UNL (27). In an Italian study, 5 of 17 (29.4%) patients were identified as having presymptomatic LOPD with isolated hyperCKemia (14). Another Spanish study revealed a 2.2% prevalence of LOPD in isolated hyperCKemia (28). Interestingly, all those presymptomatic patients were heterozygous for the leaky splice mutation c.-32-13T>G in intron 1 of *GAA* (formerly termed IVS1-13T>G) (14, 28). This common splice site mutation gives rise to alternatively spliced transcripts, including a deletion of the first coding exon 2, but still leads to residual activity of 10–15% per allele (29–31). The underlying phenotypic modifying factor and mechanism remain to be elucidated.

Another significant finding is that the eight newly diagnosed patients had respiratory involvement more frequently than in some high-risk screening studies in other populations (8, 9, 12, 15). Six patients required non-invasive ventilation at the time of diagnosis or shortly thereafter. Despite the rather late age at onset, which is an indicator of a milder phenotype, most patients still showed preferential respiratory involvement. This was consistent with the findings of a previous study reporting poor lung function in younger Chinese patients with LOPD when compared with patients globally (7). Generally, the FVC-U predictive threshold for night-time ventilation was 39% of the predicted value (32). However, hypercapnia could occur in patients with a larger ventilatory reserve, as observed in patients 2 and 6 in our study. It may involve the presence of sleep-disordered breathing and blunted ventilatory responsiveness (13, 32, 33). Respiratory parameters after ERT are less responsive than in motor outcomes but some respiratory parameters, such as the number of hours in respirator decrease, might be valuable (34–36). In the study, patient 3 reported a reduced time on the ventilator. Regarding the limited efficiency of treatment in respiratory parameters, the early diagnosis is even more important in adult Chinese LOPD, given the preferential respiratory involvement (35).

There is a significant difference in the prevalence of *GAA* IVS1 mutation in the present study in comparison to other screening studies with LOPD. The IVS1-13T>G mutation was identified with an Minor Allele Frequency (MAF) of 0.00279553 in the 1000 Genome Project (2015aug_all) but was not found in the East Asian population (EAS) (http://www.1000genomes.org.) The c.-32-13T>G variant is the most prevalent mutation accounting for approximately 40–70% of Caucasian LOPD cases (37–39). We investigated the relationship between c.-32-13T>G and respiratory involvement using data from the Pompe disease GAA variant database (http://www.pompevariantdatabase.nl.), as c.-32-13T>G is considered to be a relatively “mild” mutation. We found that the c.-32-13T>G/null compound heterozygote and c.-32-13T>G homozygote LOPD cases have less respiratory involvement than those carrying no c.-32-13T>G on any allele (*p* = 0.006, data not shown). Meanwhile, we could not establish any association between the severity of respiratory involvement and the c.2238G>C variant, the most prevalent mutation in Chinese patients with LOPD (data not shown) (16, 17, 21, 22). Therefore, predominant respiratory involvement in Chinese patients with LOPD may partly result from the extremely low prevalence of c.-32-13T>G mutation when compared with that seen in other ethnicities (40).

The analysis of clinical efficacy in adult patients demonstrated that response to ERT is widely variable in each patient (41). Due to the limited number of patients that received ERT in this study, we were not able to explore the correlations between patients' mutations and the changes in functions after treatment. Further investigation in long-term and larger Chinese cohorts is needed to explore the factors underlying this variability of clinical response to ERT (42).

## Conclusion

Herein, the LOPD prevalence was 1.6% in a high-risk Chinese population. Less frequent hyperCKemia and predominant RI depict a different early portrait of adult Chinese patients with LOPD. A modified high-risk screening strategy should be proposed to establish an early diagnosis in Chinese patients with LOPD. A larger and more systematic survey is necessary to further reveal the actual prevalence and overall perspective of LOPD in China.

## Data availability statement

The datasets presented in this study can be found in online repositories. The names of the repository and accession number can be found below: National Center for Biotechnology Information (NCBI) BioProject, PRJNA853527.

## Ethics statement

The studies involving human participants were reviewed and approved by Medical Ethics Committee of Huashan Hospital, Shanghai Medical College, Fudan University (approval no. KY2021-537). The patients/participants provided their written informed consent to participate in this study. Written informed consent was obtained from the individual(s) for the publication of any potentially identifiable images or data included in this article.

## Author contributions

WZ and QF devised the study. WZ, JD, and KJ analyzed the data, designed the graphical illustrations, and wrote the first draft of the manuscript. WZ and JD revised the manuscript. WZ oversaw the general direction of the article and critically reviewed the manuscript. All authors contributed to the project development, data collection, management, read, and approved the final version of the manuscript.

## Funding

This study was supported by the Beijing Health Promotion Association (BJHPA), China. WZ, JX, CZ, DY, and KJ were supported by the National Natural Science Foundation of China (8217052229 and 81901279); Science and Technology Commission of Shanghai Municipality (20S31904200 and 19ZR1445300); and Fuqing Scholar Student Scientific Research Program of Shanghai Medical College, Fudan University (FQXZ202106B). QF was supported by the Medical Innovation Team of Jiangsu (CXTDA2017026) and the Clinical Expert Team Introduction Project of Suzhou (SZYJTD201802).

## Conflict of interest

Author WZ has received honoraria and travel funding from Sanofi during the past 5 years. The remaining authors declare that the research was conducted in the absence of any commercial or financial relationships that could be construed as a potential conflict of interest.

## Publisher's note

All claims expressed in this article are solely those of the authors and do not necessarily represent those of their affiliated organizations, or those of the publisher, the editors and the reviewers. Any product that may be evaluated in this article, or claim that may be made by its manufacturer, is not guaranteed or endorsed by the publisher.

## Abbreviations

ACMG: American College of Medical Genetics and Genomics
CK: creatine kinase
DBS: dried blood spot
DNA: deoxyribonucleic acid
ERT: enzyme replacement therapy
FVC: forced vital capacity
FVC-U: FVC in the upright position
GAA, acid α-1: 4-glucosidase
LOPD: late-onset Pompe disease
MS/MS: tandem mass spectrometry
NGS: next-generation sequencing
UNL: upper limit of normal.
